# Conflicting results of prenatal FISH with different probes for Down's Syndrome critical regions associated with mosaicism for a de novo del(21)(q22) characterised by molecular karyotyping: Case report

**DOI:** 10.1186/1755-8166-3-16

**Published:** 2010-09-05

**Authors:** Christel Eckmann-Scholz, Stefan Gesk, Inga Nagel, Andrea Haake, Susanne Bens, Simone Heidemann, Monika Kautza, Christian Timke, Reiner Siebert, Almuth Caliebe

**Affiliations:** 1 Department of Gynecology & Obstetrics, University Hospital Schleswig-Holstein, Campus Kiel & Christian-Albrechts-University Kiel, Kiel, Germany; 2 Institute of Human Genetics, University Hospital Schleswig-Holstein, Campus Kiel & Christian-Albrechts-University Kiel, Kiel, Germany; 3 Department of Pediatrics, University Hospital Schleswig-Holstein, Campus Kiel & Christian-Albrechts-University Kiel, Kiel, Germany; 4 Praxis für Humangenetik, Kiel, Germany

## Abstract

For the rapid detection of common aneuploidies either PCR or Fluorescence in situ hybridisation (FISH) on uncultured amniotic fluid cells are widely used. There are different commercial suppliers providing FISH assays for the detection of trisomies affecting the Down's syndrome critical regions (DSCR) in 21q22. We present a case in which rapid FISH screening with different commercial probes for the DSCR yielded conflicting results. Chromosome analysis revealed a deletion of one chromosome 21 in q22 which explained the findings. Prenatally an additional small supernumerary marker chromosome (sSMC) was discovered as well, which could not be characterised. Postnatal chromosome analysis in lymphocytes of the infant revealed complex mosaicism with four cell lines. By arrayCGH the sSMC was provisionally described as derivative chromosome 21 which was confirmed by targeted FISH experiments.

## Background

Fluorescence in situ hybridisation (FISH) on uncultured amniotic fluid cells is a widely used means for the rapid prenatal diagnosis of common aneuploidies. Different commercial suppliers provide FISH assays for the detection of trisomies involving the Down syndrome critical regions (DSCR) in 21q22 which have been extensively validated in single institution series [1,2] and multicenter trials [3]. Interpretation of FISH results may be difficult if unexpected results are detected which for example can be caused by structural aberrations or mosaicism. Here we present a case in which rapid FISH screening with different commercial probes for the Down's syndrome critical regions yielded conflicting results with regard to a partial monosomy 21q. Moreover, by extensive conventional and molecular karyotyping we show this diagnostic problem to be caused by a de novo del(21)(q22) as part of a mosaic karyotype. Deletion of 21q is a rare chromosome disorder. In a recent review of 23 patients of whom reliable mapping data are available the variable phenotype depending on the deleted region became obvious [4]. Intrauterine growth retardation which was the initial presentation of the proband seems to be a constant finding.

## Results

### Case presentation

**Figure 1:**
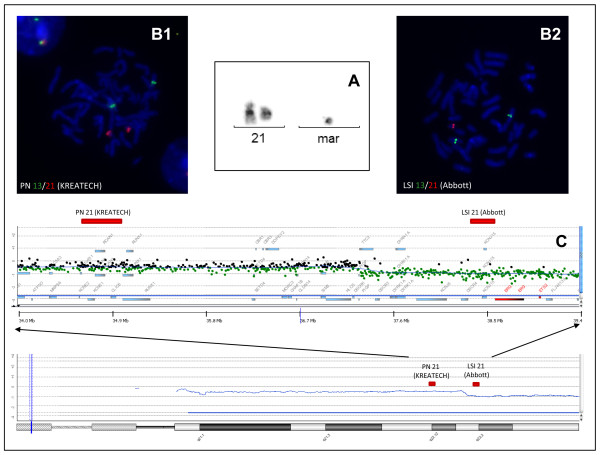
**Mapping of commercial probes.** A: Partial karyotype of the infant with del(21)(q22) and supernumerary marker chromosome. B: fluorescence-in-situ-hybridization with probes for the Down-syndrome critical regions (B1: Kreatech, B2: Abbott) showing conflicting results. C: The array profile confirms the del(21)(q22) as well as the mosaicism for the derivative chromosome 21 and the supernumerary marker chromosome originating from chromosome 21.

A 35-year-old woman presented at 24+0 weeks of gestation of her fourth pregnancy. She had suffered two early pregnancy losses. The third pregnancy ended in the delivery of a healthy boy. Medical and family history of the proposita and her partner were unremarkable. First trimester-screening including ultrasound and maternal serum biochemistry had been normal (adjusted risks +21 = 1:1839; +18 = 1:610; +13 = 1:3515). In the 25^th ^week, ultrasound revealed symmetric foetal retardation with cerebral ventriculomegaly, partial agenesis of the corpus callosum, short nasal bone and hyperechogenic bowel. Therefore, amniocentesis was performed and foetal karyotyping initiated. For rapid screening for aneuploidies, FISH was performed according to standard methods on uncultured amniotic cells using a commercially available probe set (Abbott, Wiesbaden). Signal patterns indicated a normal female gonosome constellation without evidence for aneuploidies detectable with the probes for chromosomes 13 and 18. Nevertheless, the approximately 200 kb-sized LSI21 probe for the DSCR1 containing the loci D21S529, D21S341 and D21S342 in 21q22 showed only one signal in 97 of 100 (97%) evaluated nuclei. To corroborate these findings by an independent probe, FISH was performed with a different commercial probe, PN21 (Kreatech, Berlin). This probe containing the markers D21S65, RH72110 and RH92717 and hybridising to DSCR4 and 8, revealed a normal pattern with two signals in the majority (86/100) of the nuclei, whereas a minority (14%) lacked one signal. Additional FISH analyses on uncultured amniotic cells with PAC probes for 21q11.2~21 (RP1-270M7 and RP1-152M24) as well as a commercial probe (Abbott, Wiesbaden) for the AML1 locus in 21q22 yielded a normal signal pattern. Mapping of both commercial probes indicated that they both hybridise approximately 3,3 Mb apart with the Abbott probe being located telomeric of the Kreatech probe (see Fig. 1). Therefore, the FISH patterns were judged as indicative for a de novo deletion in 21q with the breakpoint between the regions the two probes hybridise to. This interpretation was confirmed by the results of chromosome banding analysis of 15 metaphases from two independent cultures. All metaphases analysed showed a terminal deletion of the long arm of chromosome 21 with the breakpoint in 21q22. Moreover, in all metaphases an additional small marker chromosome (sSMC) was detected, of which the origin could not be identified using DA/DAPI staining and various FISH probes. The karyotype was described as 47, XX, del(21)(q22),+mar[15]. Chromosome analysis in the parents including FISH with chromosome 21 specific probes revealed a normal female respectively male karyotype in 10 metaphases analysed.

The couple was extensively counselled on the results and the pregnancy was continued. The pregnancy was monitored regularly by ultrasound. Foetal growth restriction was obvious onward. By the end of the pregnancy the patient revealed clinical signs of preeclampsia so that birth was induced at 41+2 weeks of gestation.

The child was born at 41+3 weeks with a length of 46 cm (-2.74 SD), weight of 2240 g (-4.4 SD), and a head circumference of 31 cm (-3.16 SD). APGAR scores were 1/8/9. On examination a high nasal root, down-slanting palpebral fissures, retrogenia, posterior rotated, slightly low-set ears, a long philtrum, and a thin upper vermillion were noticed. The fingernails were small. A sacral dimple was observed. Ultrasound of the abdomen gave inconspicuous results. Echocardiography showed a patent ductus artiosus Botalli (PDA) and atrial septal defect (ASD) without hemodynamic consequence. Cerebral ultrasound showed a missing septum pellucidum. The infant had a good muscle tone. The major problem was the feeding, most probably due to sucking weakness and a lack of coordination so that a feeding tube had to be placed.

To confirm the prenatal findings and further characterise the chromosomal changes DNA was extracted from cord-blood taken at birth and subjected to molecular karyotyping using two different platforms. Array CGH was performed using the Human Genome CGH Microarray 244A platform (Agilent Technologies, Santa Clara, USA). Moreover, hybridization to Cyto2.7 arrays (Affymetrix, Santa Clara, CA, USA) was performed. Both array platforms confirmed the presence of a most likely telomeric deletion with the breakpoint in 21q22 at around chr21: 37,381,170 bp (Agilent 244A) and 37,302,549 bp (Cyto2.7 Affymetrix), both mapped according to NCBI Build 36. The molecular karyotyping results explained the discrepant results with the commercial FISH probes PN21 from Kreatech (~34,538,500-35,086,000 bp) and LSI 21 from Abbott (~38,353,445-38,553,733 bp) for 21q, which hybridise centromeric and telomeric of the breakpoint, respectively. Nevertheless, in addition both array platforms provide also evidence for a deletion of 21q11.2q22.13(14,319,839-37,381,170 bp) in a subset of cells as indicated by the decrease of the signal intensity to around -0.5 as typical for mosaicism of a loss. Therefore, we performed chromosome banding analysis on a peripheral blood sample of the child which indeed yielded evidence for the presence of four cell lines differing by the presence and absence of the del(21) and the sSMC. The karyotype was described as: mos 47, XX, del(21)(q22),+mar[22]/46, XX, del(21)(q22)[7]/46, XX, -21, +mar[5]/45, XX, -21[4]. The complete loss of chromosome 21 in 9 of 38 metaphases explains the decreased copy number in 21q11.2q22.13 in the array analyses and is also in line with the prenatal FISH analyses indicating loss of 21q21~22 including DSCR4/8 in 14% of nuclei. The lack of an imbalance at the very centromeric part of 21q in the array analyses along with the absence of larger copy number gains on other chromosomes suggests, that the sSMC is derived from chromosome 21 and represents a del(21)(q11.2). To confirm this view, FISH was performed with a probe (PAC 1174A5) for the rRNA gene cluster which yielded a signal on the sSMC [5]. A weak signal was observed with a whole chromosome painting probe for chromosome 21 (Kreatech, Berlin). Hybridization with a probe detecting the centromeres of chromosomes 13 and 21 (Kreatech, Berlin) showed a signal on the sSMC. In summary these findings render the interpretation that the sSMC, is a del(21)(q11.2) likely.

## Discussion

In summary we report on a case with pre- and postnatal findings of partial monosomy 21q22 and mosaicism for the loss of the normal chromosome 21. With a commercial FISH probe binding distal to the breakpoint this gave a pattern suggestive of monosomy, whereas with a second probe binding proximal to the breakpoint the distribution of the signals was normal. Part of the complex mosaicism could have been suspected by the findings obtained on uncultivated amniotic cells with a probe binding proximal to the breakpoint in 21q22, but it was missed on the cultured amniotic cells. As no fibroblasts of the patient were analysed it cannot be ruled out that the complex mosaicism arose as a postzygotic error in blood lymphocytes.

In about 0,075% of prenatal cases an sSMC is detected [6]. Like in this case it is not always possible to characterise the sSMC on the prenatal sample due to lack of material. As the main aberration is the deletion 21q22 terminal of 37,3 Mb. The patient's deletion is similar to those described by Matsumoto et al and Yao et al [7,8] (patient 1) and Lyle et al. [9] (patient 35). The spectrum of patients with 21q deletions is wide. Lindstrand et al. [4] described three new patients and reviewed 38 patients in the literature and databases. For 20 patients reliable data of molecular or molecular cytogenetic investigations were available. According to their findings deletions involving a 0,56 Mb region between 34,796 and 35,363 kb (from pter) are associated with severe heart disease. This is in line with our findings as the patient presented only with a PDA and ASD both of no hemodynamic relevance. The region for mental retardation was narrowed down to 33,833 - 33,992 kb. The observation time in our patient is too short for any conclusions. Intrauterine growth retardation (IUGR) seems to be a constant symptom. Chromosome analysis on the patient's lymphocytes revealed a cell line with monosomy for chromosome 21. In retrospect this cell line was also present in uncultured amniotic fluid cells, therefore it represents most likely true mosaicism. Full monosomy 21 (FM21) is a rare finding and probably incompatible with life (for review see [10]). We are only aware of one live-born patient with FM21 in whom the monosomy was proven by molecular cytogenetic methods in various tissues [11]. FM21 is characterised by severe IUGR, brain malformations (holoprosencephaly, polymicrogyria), facial dysmorphism, heart defects, and severe ocular malformations. There are patients in whom mosaicism for a cell line with monosomy 21 and a second cell line for example with trisomy 21 was detected [12]. In those patients the symptoms of monosomy 21 dominated over those of trisomy 21. The proband does not have any of the severe malformations of FM21. There is some overlap in the symptoms of partial and full monosomy 21. As the percentage of cells with monosomy 21 was comparatively low in the proband this cell line did not have a detrimental effect.

## Conclusions

FISH on uncultivated amniotic cells with commercial probes is a widespread method to detect aneuploidies in prenatal diagnosis. Caution has to be warranted with regard to the interpretation of FISH patterns from prenatal screening if they do not represent the typical signal patterns. In order to interpret the results knowledge as to the precise localisation of the clones and hybridization with additional probes may be necessary. In most cases the result of FISH on uncultivated amniotic cells is in line with that of the chromosome banding analysis. However, this case highlights that structural aberrations and mosaicism can lead to results which are difficult to interpret. In general FISH for five chromosomes does not rule out numerical aberrations of all other chromosomes, structural aberrations, and sSMCs. False-positive respectively false negative results are also possible due to dicentric chromosomes, centromeric polymorphism, and maternal contamination [13]. FISH on uncultured cells has to be supplemented by conventional banding analysis. ArrayCGH is a powerful tool in the precise delineation of the structural aberration. Detection of mosaicism is possible but complex mosaicism is better characterised by techniques based on the evaluation of single cells.

## Abbreviations

PCR: polymerase chain reaction; FISH: Fluorescence in situ hybridization; DSCR: Down syndrome critical region(s); SSMC: small supernumerary marker chromosome; RRNA: ribosomal RNA; PDA: patent ductus Botalli; ASD: atrial septum defect; FM21: full monosomy 21.

## Consent

After IRB-approval informed consent of the patient is given.

## Competing interests

The authors declare that they have no competing interests.

## Authors' contributions

CES performed the ultrasound examinations, the obstetric management and drafted the manuscript, SG performed the pre- and postnatal FISH analyses, IN and SB performed array CGH on the Agilent 244K platform, AH performed molecular karyotyping on the Cyto2.7 platform, SH performed the postnatal cytogenetic analysis and helped in drafting the manuscript, MK counselled the patients on the results, CT evaluated the child postnatally, RS supervised the experiments and aligned the FISH probes, AC performed the prenatal chromosome analysis, examined the proband postnatally for dysmorphism and helped in drafting the manuscript.

All authors read and approved the final manuscript.
